# Protocol to validate custom-designed BaseScope probes using cell-free synthesized protein lysates and *in vitro*-transcribed purified mRNA

**DOI:** 10.1016/j.xpro.2025.104180

**Published:** 2025-10-30

**Authors:** Theresa Hartung, Anne Zemella, Andreas Meisel

**Affiliations:** 1 Department of Neurology with Experimental Neurology, Universitätsmedizin Berlin, Charitéplatz 1, 10117 Berlin, Germany; 2 Berlin Institute of Health at Charité, Universitätsmedizin Berlin, Anna-Louisa-Karsch Straße 2, 10178 Berlin, Germany; 3 Fraunhofer Institute for Cell Therapy and Immunology-IZI, Branch Bioanalytics and Bioprocesses-IZI-BB, Am Mühlenberg 13, Potsdam, Germany; 4 Center for Stroke Research Berlin, Neuroscience Clinical Research Center (NCRC) and Integrated Myasthenia Gravis Center, Charité - Universitätsmedizin Berlin, Charitéplatz 1, 10117 Berlin, Germany

**Keywords:** molecular biology, neuroscience, protein biochemistry, stem cells

## Abstract

BaseScope is an ultra-sensitive *in situ* hybridization technique specifically designed for detection of rare, short RNA molecules, particularly enabling co-detection of exon junctions in splice variants. Its use is cost intensive due to required protocol optimization for each tissue, especially when target expression is very low. Here, we present a protocol to validate functionality of custom-designed BaseScope probes by utilizing cell-free synthesized protein lysates and *in vitro*-transcribed purified mRNA as positive controls. We detail steps for slide preparation, probe hybridization, signal amplification, and detection.

## Highlights

• Steps for performing a duplex chromogenic BaseScope assay using cell-free liquid samples
• Procedures for validating functionality of custom-designed BaseScope probes
• Guidance on using protein lysates and *in vitro*-transcribed mRNA as positive controls
• Workflow for applying the method to liquid biopsies and RNA therapeutic validation

## Abstract (editor highlights)

Publisher’s note: Undertaking any experimental protocol requires adherence to local institutional guidelines for laboratory safety and ethics.

## Graphical abstract

**Figure:**
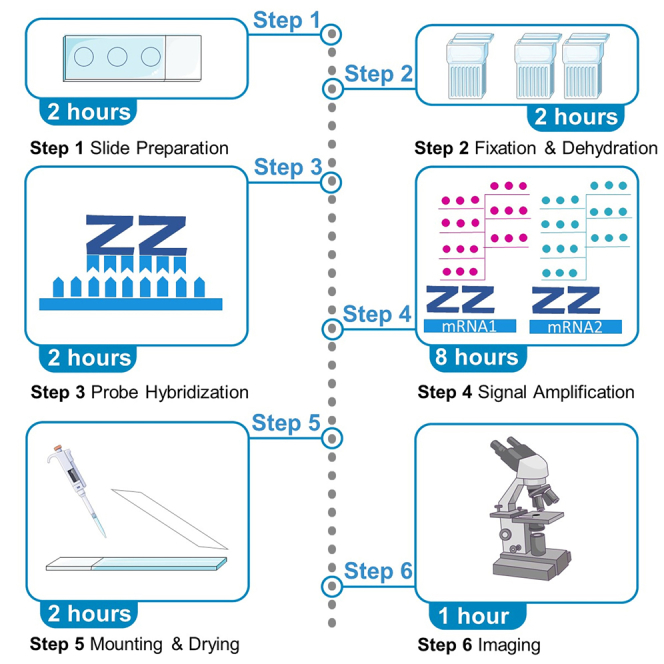

## Before you begin

We aim to detect and visualize human erythropoietin (EPO) mRNA and its splice variant hS3 mRNA in the human brain where expression is anticipated to be exceptionally rare. To date, the expression of hS3 in the human adult brain has not been verified, leaving open the possibility that it may not be expressed at all. Given the anticipated low expression levels, rigorous verification of probe specificity is particularly important. To detect EPO and hS3 mRNA, customized BaseScope probes were designed by Advanced Cell Diagnostics (ACD). As the target is a low-abundance transcript for which no validated positive control tissue or cell line is available, the primary aim was to confirm the functionality of the ACD-designed BaseScope probes. Therefore, the assay was initially performed as a qualitative evaluation to determine whether the probe produces a detectable signal (“yes” or “no”). EPO mRNA consists of five exons, while the human splice variant hS3 is characterized by the deletion of exon 3.^1^ The EPO probe targets a 38-base sequence spanning the exon 2 to exon 3 junction (GAGGCCAAGGAGGCCGAGAATATCACGACGGGCTGTGC), while the hS3 probe targets a 36-base sequence spanning the exon 2 to exon 4 junction (AGGCCAAGGAGGCCGAGAATATCACGGTCGGGCAGC). The BaseScope probes are designed as “Z-probe” pairs. The lower region of the probe binds the target mRNA, while the upper region contains a preamplifier binding site to facilitate the attachment of multiple amplifiers, thereby enhancing the chromogenic signal. The EPO probe is assigned to channel 2 (C2) and is detected using an alkaline phosphatase (AP) Fast-Red based reaction, resulting in a red signal. The hS3 probe is assigned to channel 1 (C1) and is detected using a horseradish peroxidase (HRP)-based reaction, producing a green signal. Depending on the target, the functionality, sensitivity and specificity of custom-designed BaseScope probes can be validated, for example by using cell lines that specifically express the target transcript^2^ or by generating a mouse strain expressing only the variant of interest.^3^ However, generating such cell lines or mouse strains can be technically challenging, time-consuming, and cost-intensive. To validate the functionality of our EPO and hS3 customized BaseScope probes we developed a BaseScope protocol for cell-free synthesized EPO and hS3 protein lysates and in vitro transcribed purified EPO and hS3 mRNA as positive controls before implementing the BaseScope assay in human brain samples. BaseScope protocols have been established for tissue and cells, more precisely fresh-frozen sections, formalin-fixed paraffin-embedded (FFPE) sections and cultured cells.^4^ To our knowledge, we are the first to establish a duplex chromogenic BaseScope protocol for cell-free liquids. Cell-free protein synthesis offers a highly efficient alternative to time-consuming cell-based protein production and has recently been optimized for EPO.^5^ In cell-free protein synthesis, all components required for transcription and translation are utilized without the need for a cellular membrane or living cells, with both processes occurring simultaneously in a single reaction tube. The resulting mRNA can be purified via gel filtration. The protein lysate consists exclusively of the microsomal fraction, as this is expected to contain proteins with appropriate post-translational modifications.^6^ It is hypothesized that mRNA adheres to the outer surface of the microsomes and is co-isolated during microsomal centrifugation. Upon disruption of the microsomes, the mRNA may be released and co-elute with the proteins into the supernatant. However, this remains a hypothesis and requires further experimental validation. Regardless of the underlying mechanism, cell-free synthesized EPO and hS3 protein lysates consistently retain amounts of mRNA, enabling detection by BaseScope. Similarly, assay establishment was also feasible using in vitro-transcribed mRNA purified by gel filtration, which is generated as part of the cell-free protein synthesis process.^5^ Our BaseScope protocol for cell-free liquids is based on the Sample Preparation Technical Note for Cultured Adherent Cells using RNAscope 2.5 Chromogenic Assay (https://acdbio.com/system/files_force/321232_Tech%20Note%20CulturedCells_Chromogenic_%2011_2_1.pdf?download=1) and the BaseScope Duplex Detection manual for FFPE samples (https://acdbio.com/sites/default/files/323800-USM_BaseScope%20Duplex%20Detection%20Reagent%20User%20Manual_0.pdf). Since the sample consists neither of tissue nor cells, pre-treatment with target retrieval, H_2_O_2_, protease, rehydration or counter staining is not required. Additionally, adapted protocol sections have been modified.

### Innovation

This protocol presents the first implementation of a duplex chromogenic BaseScope assay for cell-free, mRNA-containing solutions and introduces a novel approach to validating the functionality of custom-designed BaseScope probes targeting rare transcript variants. Traditionally, probe validation requires target-expressing cell lines or tissue samples, which are often unavailable for rare transcripts and splice variants, and their generation is both time-consuming and cost-intensive. Our method overcomes these limitations by employing cell-free synthesized protein lysates and in vitro–transcribed purified mRNA as positive controls. This enables qualitative confirmation of probe functionality (“yes” or “no”) without the need for complex cellular systems.

The protocol also incorporates optimizations for handling liquid samples, including modified fixation, hybridization, and amplification steps, and provides troubleshooting strategies specific to cell-free contexts. Furthermore, it demonstrates assay sensitivity across a dilution series and offers preliminary insight into potential supramolecular RNA interactions, contributing to a deeper understanding of assay behavior in solutions.

This approach significantly reduces technical effort, turnaround time, and resource requirements, while maintaining high sensitivity and specificity. It allows rapid generation of reliable positive controls and supports validation of probes targeting rare or previously unverified transcripts. Ultimately, this proof-of-principle protocol expands the applicability of BaseScope technology beyond tissue and cell samples, offering a versatile and accessible tool for RNA detection studies, splice variant characterization, and future applications in liquid biopsy analysis or RNA therapeutic validation.

### Prepare 1× wash buffer

**Timing: 30 min**

1. Prewarm 60 mL RNAScope 50× Wash Buffer in a water bath for 20 min at 40°C.
2. Add the 60 mL prewarmed buffer to 3.2 L distilled water and mix well.

***Note:*** Wash Buffer is stable at room temperature (20°C–25°C) for 4 weeks.

### Prepare 200 mL 5× saline-sodium citrate storage buffer

**Timing: 15 min**

3. Add 50 mL 20× SSC to 150 mL distilled water.

***Note:*** Storage Buffer is stable at 4°C for 4 weeks.

### Prepare 250 mL 4% paraformaldehyde

**Timing: 2 h**

4. Dissolve 10 g of PFA in 200 mL of distilled water with one NaOH pellet at 60°C under continuous stirring on a magnetic stirrer until the solution becomes clear.
5. Separately, dissolve 2.45 g of sodium phosphate dibasic dihydrate in 50 mL of distilled water and add it to the PFA solution.
6. Allow the mixture to cool to room temperature (20°C–25°C).
7. Adjust pH to 7.4 by gradually adding 25% HCl.
8. Filter the solution through a 0.45 μm bottle-top filter.

***Note:*** PFA is stable at 4°C for 3 months.

### Prepare probe mixture

**Timing: 15 min**

9. The C1 probe (hS3) is supplied in a dropper bottle containing 3 mL. The dropper insert is carefully removed from the dropper bottle to allow for pipette-based withdrawal.
10. The C2 probe (EPO) is supplied at 50× concentration in a tube containing 60 μL. Briefly centrifuge the C2 probe to ensure the liquid is collected at the bottom of the tube.
11. Prepare the probe mixture by combining 1 volume of the C2 probe with 50 volumes of the C1 probe.

***Note:*** Probe mixture is stable at 4°C for 6 months. For each sample, 25 μL of probe mix is required.

### Prepare dehydration solutions

**Timing: 15 min**

12. Prepare 200 mL of 50% EtOH by adding 100 mL 100% EtOH to 100 mL distilled water.
13. Prepare 200 mL of 70% EtOH by adding 140 mL 100% EtOH to 60 mL distilled water.

***Note:*** Dehydration solutions are stable at 4°C for 4 weeks.

### Prepare lysate dilutions and mRNA dilutions

**Timing: 30 min**

14. Prepare 25 μL of each lysate diluted 1:2 in distilled water.
15. Prepare 25 μL of each mRNA diluted to a concentration of 20 ng/μL in distilled water.

***Note:*** Dilutions should be prepared freshly, direct before starting the protocol.

## Key resources table

**Table:** 

REAGENT or RESOURCE	SOURCE	IDENTIFIER
**Chemicals, peptides, and recombinant proteins**		

20× SSC buffer molecular biology grade	Serva	Cat# 42555.01
Paraformaldehyde	Roth	Cat# 0335.2
Sodium phosphate dibasic dihydrate	Sigma-Aldrich	Cat# 30412-1kg
Natriumhydroxide pellets	Carl Roth	Cat# 6771.1
Ethanol natriumhydroxide pellets absolute 99.9%	Baker	Cat# 15578454
Poly-D-lysine	Gibco	Cat# A3890401
Cell-free synthesized EPO Lysat	Zemella et al.^5^	N/A
Cell-free synthesized hS3 Lysat	Frauenhofer, IZI-BB	N/A
*In vitro*-transcribed EPO mRNA	Frauenhofer, IZI-BB	N/A
*In vitro*-transcribed hS3 mRNA	Frauenhofer, IZI-BB	N/A

**Critical commercial assays**		

BaseScope duplex reagent kit intro	ACDBio	Cat# 323870

**Oligonucleotides**		

BaseScope Probe BA-Hs-EPO-E2E4-C1	ACDBio, This paper	Cat# 1086901-C1
BaseScope Probe BA-Hs-EPO-E2E3-C2	ACDBio	Cat# 1086891-C2

**Other**		

HybEZ II hybridization system/oven	ACDBio	Cat# 321711 or 321721
Superfrost Plus adhesion microscope slides	Epredia	Cat# J1800AMNZ
Coverslips, 24 × 60 mm, 0.26–0.29	R.Langenbrick	Cat# 1-2460/S
RNase AWAY	Molecular BioProducts	Cat# 7000
Nuclease-free water	QIAGEN	Cat# 129115
Nalgene Rapid-Flow sterile disposable bottle top filters, 0,45 μm	Thermo Fisher Scientific	Cat# 295-4545
VectaMount permanent mounting medium	Vector Labs	Cat# H-5000
Leica DMRA2 microscope DCM6200 camera	Leica	No longer for sale

## Materials and equipment

**Table:** 4% Paraformaldehyde

Reagent	Final concentration	Amount
Paraformaldehyde	4%	10 g
distilled water	N/A	200 mL
Sodium Phosphate Dibasic Dihydrate	55 mM	2.45 g
distilled water	N/A	50 mL
Natriumhydroxide pellets	N/A	1
HCl	25%	Variable dependent on pH

Store at 4°C for 3 months.

**CRITICAL:** Paraformaldehyde (PFA) is toxic and a potential carcinogen and releases harmful formaldehyde vapors. Handle in a fume hood with gloves, lab coat, and safety goggles. Avoid inhalation and skin contact; dispose of as formaldehyde waste.

***Alternatives:*** This protocol employs cell-free synthesized EPO and hS3 lysates, EPO and hS3 in vitro-transcribed mRNA, in combination with customized EPO and hS3 BaseScope probes. Additionally, negative control lysate and negative control mRNA were included, generated through standard translation reactions without the addition of mRNA, respectively generated through standard transcription reaction without the addition of DNA (no-template control, NTC). Both approaches are adaptable to any gene of interest, including splice variants or genes harboring single nucleotide mutations. The customized cell-free synthesized lysates and in vitro-transcribed mRNA were produced by the Fraunhofer Institute for Cell Therapy and Immunology, Branch Bioanalytics and Bioprocesses-IZI-BB, Potsdam, Germany. The customized BaseScope probes were designed by Advanced Cell Diagnostics (ACDBio), USA. Any bright-field microscope can be utilized for imaging.

***Note:*** The efficiency of cell-free synthesis may vary depending on the gene of interest. Consequently, RNA concentrations in lysates may not correlate linearly with protein concentrations. Therefore, for qualitative verification dilution with nuclease-free water was performed independently of protein concentration 1:2 which corresponds to a protein concentration of 1 ng/μL (EPO) and 1,6 ng/μL (hS3). Optimizing the dilution of cell-free synthesized protein lysates may be necessary, as the efficiency of cell-free synthesis and the retention of RNA in the lysate may vary depending on the gene of interest. For users applying this protocol to other targets, we recommend performing a small-scale dilution series (e.g., undiluted, 1:2, 1:4) to empirically determine the optimal dilution. Up to three samples can be fixed on one microscope slide.

## Step-by-step method details

### Preparation of microscope slides—Day 1

**Timing: 2 h**

This step prepares microscope slides to provide a stable and RNase-free surface for sample application. Cleaning with RNase AWAY and coating with Poly-D-Lysine ensure proper sample adhesion and minimize RNA degradation. Creating hydrophobic barriers defines reaction areas, allowing controlled application of lysate or mRNA droplets. Proper preparation at this stage is critical for uniform fixation and consistent hybridization results in later steps.

1. Prepare Superfrost microscope slides under a fume hood.
  a. Cover Superfrost microscope slides with RNase AWAY.
  b. Incubate for 5 min.
  c. Wipe dry using Kimwipe.
  d. Apply up to three 1 cm diameter hydrophobic barriers on each slide using the ImmEdge Hydrophobic Barrier PAP Pen (included in the BaseScope Duplex Reagent Kit Intro) to accommodate up to three samples.
  e. Allow the barriers to completely dry under the fume hood.

**CRITICAL:** Make sure the hydrophobic barrier is completely dry before proceeding.

2. Dilute Poly-D-Lysine 1:1 with 1× PBS.
  a. Add 50 μL to each hydrophobic barrier circle.
  b. Incubate the slide for 1 h at room temperature (20°C–25°C) covered with a lid, such as a standard cardboard lid from a tube storage box.
  c. Immerse the slide three times in distilled water and allow the slide to dry under the fume hood.
  d. Apply 25 μL of pre-diluted lysate or mRNA by dispensing it dropwise in the center of the circle using a 100 μL pipette.
  e. Allow the sample to dry at room temperature (20°C–25°C) under the fume hood covered with a lid, such as a standard cardboard lid from a tube storage box for approximately 45 min.

**Figure 1:**
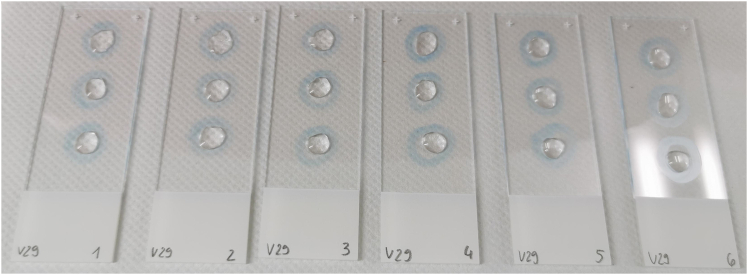
Photograph of microscope slide preparation setup

***Note:*** Droplets placed on the slide within a reaction field of approximately 1 cm in diameter do not dry out after 1 h of incubation under a cover but remain unchanged. A photograph of the setup is provided in Figure 1.

### Fixation and dehydration of liquid samples—Day 1

**Timing: 2 h**

This step fixes and stabilizes the liquid samples on the prepared slides to preserve RNA integrity during hybridization and amplification. Paraformaldehyde fixation crosslinks RNA molecules, preventing diffusion or degradation, while graded ethanol dehydration removes residual moisture and enhances probe accessibility. Proper fixation and dehydration are essential to maintain the structure of the droplet and prevent RNA loss during subsequent incubations.

***Note:*** For liquid samples, prolonged fixation and dehydration are required to ensure stability.

3. Fix the slides in 4% PFA for 1 h at room temperature (20°C–25°C) using a glass staining jar.
4. Wash slides twice in 1× PBS using a glass staining jar.
5. Dehydrate slide in 50% EtOH for 5 min using a glass staining jar.
6. Dehydrate slide in 70% EtOH for 5 min using a glass staining jar.
7. Dehydrate slide in 100% EtOH for 5 min using a glass staining jar.
8. Dehydrate slide in fresh 100% EtOH for 10 min using a glass staining jar.
9. During dehydration, the dye from the hydrophobic barrier pen is known to dissolve; however, the outline remains clearly visible. Optionally retrace hydrophobic barrier and allow them to dry completely.

**CRITICAL:** Make sure the hydrophobic barrier is completely dry before proceeding.

### Hybridization of pre-prepared probe mixture—Day 1

**Timing: 2 h**

This step enables the hybridization of custom-designed BaseScope probes to their complementary RNA targets within the fixed liquid samples. Incubation in the HybEz oven at 40°C ensures optimal binding efficiency and specificity, while washing steps remove excess unbound probes. Proper hybridization is critical to achieving accurate and sensitive detection of target RNA molecules during the amplification phase.

***Note:*** For liquid samples, prolonged hybridization is required to ensure sufficiency.

10. Hybridize probes using the HybEz oven.
  a. Turn on the HybEz oven and set it to 40°C (RNAscope mode).
  b. Place slides in the plastic EZ-Batch Slide Holder, supplied with the HybEz oven.
  c. Apply 25 μL of probe mixture to each sample by dispensing it dropwise in the center of the circle using a 100 μL pipette.
  d. Insert the Slide Holder into the aluminum humidity control tray supplied with the HybEz oven.
  e. Incubate slides for 2 h at 40°C.

**CRITICAL:** The insertion of the slide holder into the aluminum tray and its placement in the oven must be performed strictly horizontally to prevent the droplet from overflowing beyond the hydrophobic barrier.

11. Wash slides to remove excess hybridized probes.
  a. Pour 200 mL wash buffer in the EZ-Batch Wash Tray supplied with the HybEz oven.
  b. Place plastic EZ-Batch Slide Holder with slides in the wash tray containing washing buffer and make sure that the slide is submerged.
  c. Wash slides with slight rocking agitation for 2 min.
  d. Repeat washing step once with fresh washing buffer.
  e. Stopping Point (optional, recommended), store slide in 5× SSC buffer in a light-tight jar overnight (12–18 h) at room temperature (20°C–25°C).

***Note:*** All washing steps are conducted at room temperature (20°C–25°C).

### Signal amplification and detection—Day 2

**Timing: 8 h**

This step amplifies and visualizes the hybridized probe signals to allow chromogenic detection of target RNA molecules. Sequential application of AMP reagents builds a branched amplification structure that enhances signal intensity, followed by enzyme-mediated chromogenic reactions producing distinct red and green signals for each probe channel.

***Note:*** The incubation time of the amplification steps has been modified to increase signal detection. All incubation steps at 40°C are performed in the HybEz oven with slides placed in the plastic EZ-Batch Slide Holder and inserted into the aluminum humidity control tray.

**CRITICAL:** The insertion of the slide holder into the aluminum tray and its placement in the oven must be performed strictly horizontally to prevent the droplet from overflowing beyond the hydrophobic barrier. Do not let the slides dry out throughout the assay. Following every two wash steps with wash buffer, the washing tray is rinsed with distilled water and wiped dry.

12. Prepare slides for signal amplification.
  a. Let amplification reagents (AMP 1–12) adapt to room temperature (20°C–25°C).
  b. Turn on the HybEz oven and set it to 40°C (RNAscope mode).
  c. Place humidifying paper in the aluminum humidity control tray and wet it with distilled water.
  d. Wash slides twice for 2 min in 200 mL washing buffer to remove SSC buffer.
  e. Remove excess liquid by flicking the slides 3–5 times.
13. Signal amplification of C2 probe (EPO).
  a. Apply one drop of AMP 1 in the middle of each sample and incubate for 30 min at 40°C.
  b. Wash slides twice for 2 min in 200 mL washing buffer and remove excess liquid by flicking the slides 3–5 times.
  c. Apply one drop of AMP 2 in the middle of each sample and incubate for 30 min at 40°C.
  d. Wash slides twice for 2 min in 200 mL washing buffer and remove excess liquid by flicking the slides 3–5 times.
  e. Apply one drop of AMP 3 in the middle of each sample and incubate for 15 min at 40°C.
  f. Wash slides twice for 2 min in 200 mL washing buffer and remove excess liquid by flicking the slides 3–5 times.
  g. Apply one drop of AMP 4 in the middle of each sample and incubate for 30 min at 40°C.
  h. Wash slides twice for 2 min in 200 mL washing buffer and remove excess liquid by flicking the slides 3–5 times.
  i. Apply one drop of AMP 5 in the middle of each sample and incubate for 30 min at 40°C.
  j. Wash slides twice for 2 min in 200 mL washing buffer and remove excess liquid by flicking the slides 3–5 times.
  k. Apply one drop of AMP 6 in the middle of each sample and incubate for 15 min at 40°C.
  l. Wash slides twice for 2 min in 200 mL washing buffer and remove excess liquid by flicking the slides 3–5 times.
  m. Apply one drop of AMP 7 in the middle of each sample and incubate for 60 min at room temperature.
  n. Wash slides twice for 2 min in 200 mL washing buffer and remove excess liquid by flicking the slides 3–5 times.
  o. Apply one drop of AMP 8 in the middle of each sample and incubate for 15 min at room temperature.
14. Detect the red signal.
  a. During incubation, prepare the Fast RED mixture by combining 1 volume of RED B with 60 volumes of RED A.
  b. For each sample, 25 μL of the mixture is required. Prepare the amount needed for all samples in one tube at once. Mix well and protect mixture from light exposure.
  c. Wash slides twice for 2 min in 200 mL washing buffer and remove excess liquid by flicking the slides 3–5 times.
  d. Apply 25 μL Fast Red mixture on each sample and incubate for 10 min at room temperature (20°C–25°C) protected from light exposure.
  e. Wash slides twice for 2 min in 200 mL washing buffer and remove excess liquid by flicking the slides 3–5 times.

**CRITICAL:** Use the Fast Red mixture within 5 min. Do not expose to direct sunlight or UV light.

15. Signal amplification of C1 probe (hS3).
  a. Apply one drop of AMP 9 in the middle of each sample and incubate for 15 min at 40°C.
  b. Wash slides twice for 2 min in 200 mL washing buffer and remove excess liquid by flicking the slides 3–5 times.
  c. Apply one drop of AMP 10 in the middle of each sample and incubate for 15 min at 40°C.
  d. Wash slides twice for 2 min in 200 mL washing buffer and remove excess liquid by flicking the slides 3–5 times.
  e. Apply one drop of AMP 11 in the middle of each sample and incubate for 60 min at room temperature (20°C–25°C).
  f. Wash slides twice for 2 min in 200 mL washing buffer and remove excess liquid by flicking the slides 3–5 times.
  g. Apply one drop of AMP 12 in the middle of each sample and incubate for 15 min at room temperature (20°C–25°C).
16. Detect the green signal.
  a. During incubation, prepare the GREEN mixture by combining 1 volume of GREEN B with 50 volumes of GREEN A.
  b. For each sample, 25 μL of the mixture is required. Prepare the amount needed for all samples in one tube at once. Mix well and protect mixture from light exposure.
  c. Wash slides twice for 2 min in 200 mL washing buffer and remove excess liquid by flicking the slides 3–5 times.
  d. Apply 25 μL GREEN mixture on each sample and incubate for 10 min at room temperature (20°C–25°C) protected from light exposure.
  e. Wash slides once for 5 min in 200 mL washing buffer.
  f. Briefly immerse the slides in distilled water and remove excess liquid by flicking the slides 3–5 times.
  g. Let slides dry in the HybEz oven at 60°C (bake mode) for 30–60 min.

**CRITICAL:** Make sure the slides are completely dry before proceeding.

### Mounting and drying—Day 2

**Timing: 2 h**

This step seals and preserves the stained samples to ensure long-term stability and high-quality imaging. Application of mounting medium and careful placement of the coverslip prevent air bubble formation and preserve chromogenic signals. Complete drying in the oven ensures optimal optical clarity and prevents sample movement during microscopy.

***Note:*** Mounting has a significant impact on image quality. We recommend practicing dry mounting immediately before mounting the samples to enhance results.

17. Add 1–2 drops of VectaMount Permanent Mounting Medium directly in the middle of each sample using a 1000 pipette.
18. Gently aspirate air bubbles using a 10 μL pipette.
19. Place the coverslip by holding it with tweezers and gradually lowering it from one side to the other.
20. Turn Oven off and let slides dry inside overnight to ensure the mounting medium is thoroughly dried before imaging.

### Image acquisition—Day 3

**Timing: 1 h**

This step documents the assay results through microscopic imaging of chromogenic signals. Capturing images soon after mounting ensures consistent signal intensity and prevents fading over time. Proper imaging settings and magnification allow clear visualization of individual RNA puncta, facilitating qualitative assessment of probe performance and overall assay success.

***Note:*** The chromogenic stain from the BaseScope assay is stable for approximately 3 months, but for comparable accuracy we recommend imaging within 4 weeks.

21. Any bright-field microscope can be utilized for imaging. We used a Leica DMRA2 with a DCM6200 camera.

## Expected outcomes

**Figure 2:**
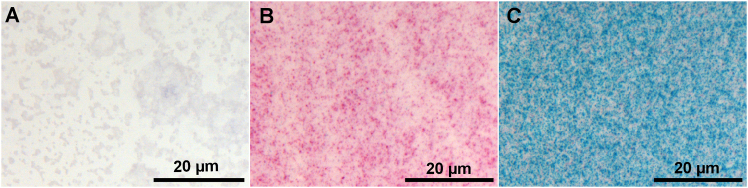
Expected outcomes – Cell-free synthesized protein lysates Confirmation of functionality of customized BaseScope probes through the detection of EPO and splice variant hS3 mRNA in cell-free synthesized lysates: (A) Negative control, (B) EPO, (C) hS3 at 20× magnification. Scale bar: 20 µm.

**Figure 3:**
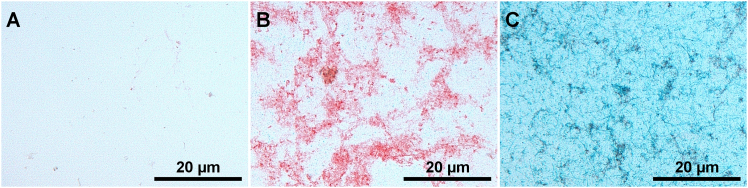
Expected outcomes – In vitro-transcribed mRNA Confirmation of functionality of customized BaseScope probes through the detection of EPO and splice variant hS3 mRNA in vitro transcribed pure mRNA: (A) Negative control, (B) EPO, (C) hS3 at 20× magnification. Scale bar: 20 µm.

Each individual EPO RNA transcript appears as a distinct red chromogen precipitate dot, while each hS3 splice variant RNA transcript is visualized as a green chromogen precipitate dot. The EPO lysate is expected to produce a red signal (Figure 2B), whereas the hS3 splice variant lysate is expected to produce a green signal (Figure 2C). In contrast, the negative control should yield no signal (Figure 2A). Similar outcomes are expected for purified in vitro-transcribed mRNA. EPO mRNA molecules produce a red signal (Figure 3B), hS3 mRNA molecules produce a green signal (Figure 3C) and in contrast negative control yields no signal (Figure 3A).

**Figure 4:**
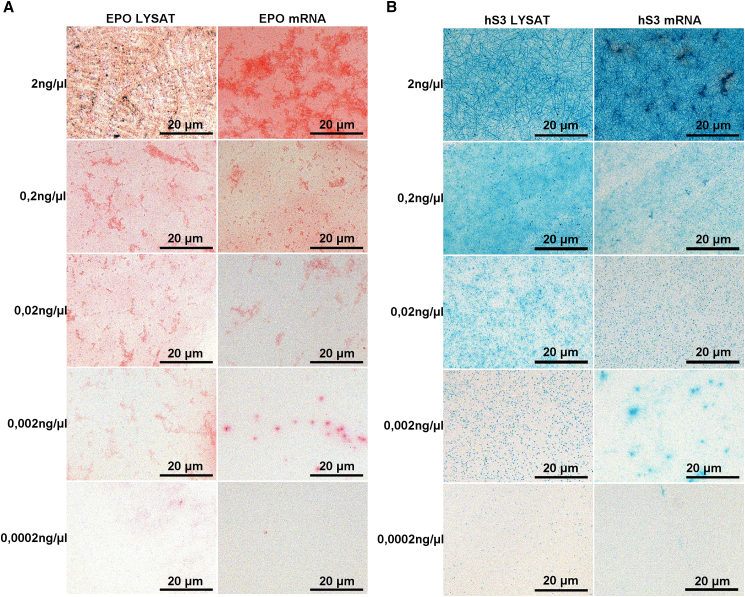
Dilution of cell-free synthesized lysates and in vitro-transcribed mRNA Dilution of cell-free synthesized lysates with protein concentrations of 2 ng/μL, 0.2 ng/μL, 0.02 ng/μL, 0.002 ng/μL and 0.002 ng/μL and dilution of in vitro-transcribed mRNA with RNA concentrations of 2 ng/μL, 0.2 ng/μL, 0.02 ng/μL, 0.002 ng/μL and 0.002 ng/μL: (A) EPO, (B) hS3 at 20× magnification. Scale bar: 20 µm.

Although the primary aim of this assay was to qualitatively confirm probe functionality, serial dilutions of the target mRNA can provide additional information on probe performance, offering an approximate indication of sensitivity through the lowest concentration yielding a detectable signal, and of specificity by assessing whether signal intensity decreases proportionally with target dilution and approaches background levels in low-concentration samples. To at least preliminary address sensitivity and specificity, we additionally performed a dilution experiment. We diluted both, the EPO (Figure 4A, left panels) and hS3 (Figure 4B, left panels) protein lysates with protein concentrations ranging from 2 ng/μL down to 0,0002 ng/μL and the EPO (Figure 4A, right panels) and hS3 (Figure 4B, right panels) in vitro transcribed mRNA with mRNA concentrations ranging from 2 ng/μl down to 0.0002 ng/μl.

Despite this experiment being limited to a single replicate, the results indicate that fibril formation is more likely to occur at higher concentrations, that protein and RNA concentrations do not correlate directly in lysates, and therefore RNA can be considered performing more robustly than protein lysates, and that low RNA concentrations as little as 0.002 ng/μl remained detectable.

Overall, this protocol offers reduced technical effort, shorter turnaround times, and the ability to rapidly generate reliable positive controls, thereby facilitating probe validation for low-abundance and splice variant targets.

***Note:*** For additional dilution experiments, new lysates and mRNA had to be ordered. Variations in dilutions and staining outcomes across different batches may arise from multiple factors. First, the efficiency of cell-free protein synthesis and in vitro transcription can differ between production batches, leading to fluctuations in the relative abundance of RNA in the lysates. Second, during the course of this work, the Fraunhofer Institute was required to change the supplier of their T7 polymerase because the original supplier discontinued production. They have consistently observed reduced protein yields when using the alternative T7 polymerase. These batch-dependent differences in transcriptional and translational efficiency likely contribute to variability in staining outcomes, not only due to altered RNA and protein yields, but also because changes in the ratio of nucleic acids or proteins to buffer may influence the drying dynamics of droplets and thereby affect staining behavior.

## Limitations

The primary technical challenge with liquid samples is maintaining RNA stability since the absence of cell boundaries permits extensive RNA–RNA interactions, making the molecules highly sensitive to environmental factors such as pH fluctuations. However, low RNA concentrations represent a second challenge, particularly in clinical liquid biopsy samples, where transcript abundance is expected to be very limited. Initial efforts in adapting a BaseScope in situ hybridization protocol for use with liquid biopsies were reported by Kirby et al.^7^ who detected circular RNAs in rat serum and amniotic fluid using the BaseScope RED Assay. Although liquid samples were successfully fixed and detection was achieved via a single red fluorescence signal, the signal intensity was insufficient to generate a clear chromogenic readout and allow quantification. In contrast, we present the first successful implementation of a duplex chromogenic BaseScope protocol for, mRNA-containing liquids, designed as a quantitative positive control for validating custom-designed BaseScope probes targeting rare mRNA species. The assay enables visualization of each individual mRNA molecule as a punctuate dot under a standard bright-field microscope. However, since mRNA concentrations in lysates and in vitro–transcribed solutions are substantially higher than in cells, and because the absence of cell boundaries permits enhanced RNA–RNA interactions, these molecules are highly sensitive to environmental factors, including pH fluctuations. Consequently, even minor variations may induce supramolecular polymer formation, e.g. fibrils.^8^ Indeed, dilution series demonstrate that fibril formation occurs predominantly at higher concentrations, where the lower proportion of RNase-free water may accelerate drying and thereby promote aggregation. Notably, fibril formation was also observed in a small central region of droplets from samples diluted down to 0,02 ng/μL. This distribution pattern suggests that, as drying proceeds from the periphery toward the center, local concentration gradients develop, leading to accumulation and subsequent fibril formation in the middle. Such heterogeneity further indicates that a reliable quantitative analysis of the dilution series is currently not feasible, e.g. only feasible for dilutions below 0.002 ng/μL. Interestingly, in our preliminary single-replicate experiment, we observed that heterogeneity may no longer be present at dilutions of and below 0.002 ng/μl, suggesting that low-input conditions could provide more homogeneous results. While dehydration and pH variation are likely contributing triggers, additional studies will be required to elucidate the mechanisms driving fibril formation in the context of this assay.

The manufacturer does not provide quantitative sensitivity specifications for BaseScope probes. In our experiments, the assay produced a clear positive signal at a protein concentration of 0.002 ng/μl in cell-free lysates and at an equivalent RNA concentration in in vitro–transcribed mRNA, whereas no sufficient signal was detected at the lowest tested concentration of 0.0002 ng/μl. Although direct comparison to manufacturer-defined thresholds is not possible, these results suggest that the assay is capable of detecting targets at low RNA input levels under the present experimental conditions.

A limitation of the present work is that we did not perform a systematic validation of sensitivity and specificity using concentration–response curves. Such validation would indeed add value, but it would likely need to be performed individually for each new target. Accordingly, we position this work as a proof-of-principle protocol demonstrating the feasibility of applying the BaseScope assay to cell-free mRNA-containing samples, rather than as a comprehensive validation.

Nevertheless, our preliminary findings provide initial indications of both sensitivity and specificity and may serve as a foundation for more systematic investigations in the future.

## Troubleshooting

### Problem 1

No staining potentially due to insufficient fixation.

### Potential solution

During protocol optimization, we evaluated alternative basement matrices for fixation, including undiluted poly-D-lysine, poly-D-lysine diluted 1:1 in 1× TAE buffer, and Geltrex, but all yielded inferior results compared to Poly-D-lysine diluted 1:1 in PBS. In cases where staining is absent, potentially due to insufficient fixation, we recommend extending the drying time to 60 min under a fume hood without covering the samples (preparation of microscope slides—day 1, Step 2e). It should be noted that additional basement matrices, such as Matrigel or Laminin, were not evaluated in this study.

### Problem 2

Extensive Fiber formation.

### Potential solution

Consider diluting samples further down to a minimum concentration of 0,002 ng/μL of protein concentration in lysates or RNA concentrations of in vitro-transcribed mRNA. Dilute with RNase free water (prepare lysate dilutions and mRNA dilutions – steps 14 / 15).

### Problem 3

Individual RNA molecules are not distinguishable as punctate dots despite sufficient dilution.

### Potential solution

**Figure 5:**
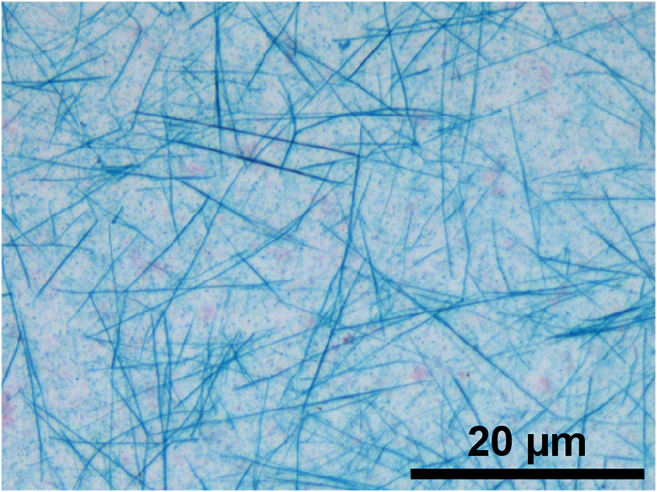
Example of RNA self-assembly into fiber-like structures. Scale bar: 20 µm.

During protocol optimization, we tested baking lysates for up to 60 min at 60°C prior to fixation. The results indicate that RNA molecules formed fibril-like structures (Figure 5). The baking step induces severe dehydration, which can lead to a decrease in pH, thereby promoting RNA protonation, which may facilitate RNA fiber formation. Cox et al.^8^ demonstrated that protonated RNA assembles into twisted fibers at room temperature.

In case fiber formation occurs, reduce the drying time (preparation of microscope slides—day 1, step 2e) and ensure that samples are not excessively dehydrated.

### Problem 4

Non-specific staining along the edge of the hydrophobic barrier due to reagent trapping.

### Potential solution

When retracing the hydrophobic barrier pen (fixation and dehydration of liquid samples—day 1, step 9) ensure it is completely dry before proceeding. Incomplete drying may cause the upper layer of the retraced barrier to partially lift from the underlying layer, forming a pocket that can trap reagents. To avoid this, either incubate slide at 40°C for at least 10 min or proceed without retracing. The transparent outline of the initial hydrophobic barrier remains visible and is sufficient to prevent reagent overflow; retracing primarily serves to enhance visual contrast through its blue coloration.

### Problem 5

Unequal sizes of hydrophobic barrier reaction fields, which would is relevant for systematic validation.

### Potential solution

**Figure 6:**
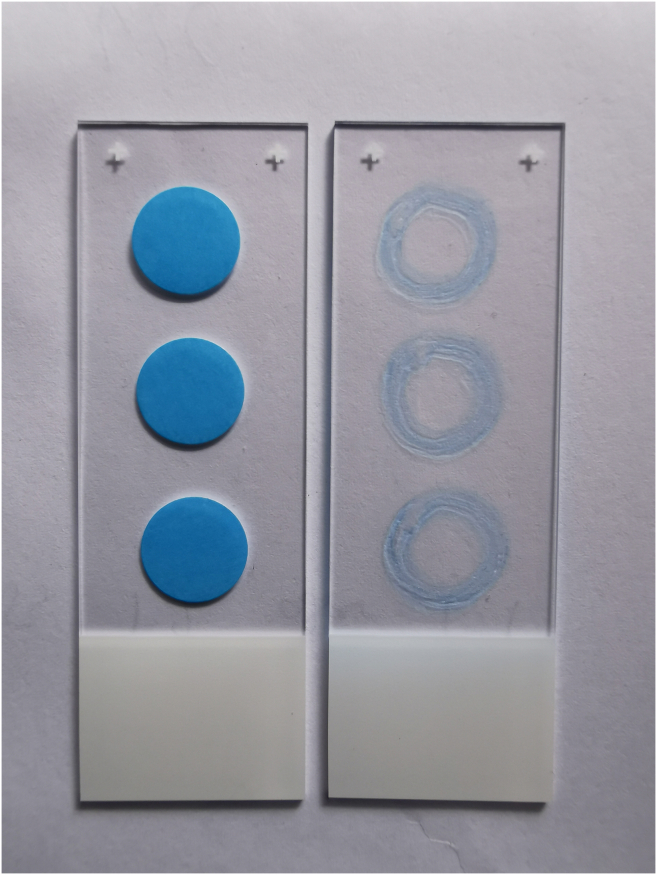
Photograph of reaction field coating on microscope slides

Prepare a template slide by affixing 3 × 12 mm adhesive labels to a microscope slide. Use this slide as a stencil by placing it underneath a clean microscope slide and tracing the edges of the circular labels with a hydrophobic barrier pen (preparation of microscope slides—day 1, Step 1d). This approach allows for the creation of reaction fields of uniform size. Alternatively, slides with prefabricated reaction wells, such as Marienfeld M03-1216490, could be tested in the assay. A photograph of the setup is provided in Figure 6.

## Resource availability

### Lead contact

Further information and requests for resources and reagents should be directed to and will be fulfilled by the lead contact Dr. med. Theresa Hartung (theresa.hartung@charite.de).

### Technical contact

Questions about the technical specifics of performing the protocol should be directed to and will be fulfilled by the technical contact, Dr. med. Theresa Hartung (theresa.hartung@charite.de).

### Materials availability

Cell-free synthesized lysates and vitro transcribed pure mRNA were produced by the Fraunhofer Institute for Cell Therapy and Immunology-IZI, Branch Bioanalytics and Bioprocesses-IZI-BB, Potsdam, Germany and can be custom-ordered upon request through Dr. Anne Zemella. BaseScope probes were designed by Advanced Cell Diagnostics (ACDBio) and deposited to their catalog (BaseScope Probe- BA- Hs-EPO-E2E3-C2 catalog number 1086891-C2, BaseScope Probe- BA-Hs-EPO-E2E4-C1 catalog number 1086901-C1).

### Data and code availability

This study did not generate a dataset or code.

## Acknowledgments

The authors gratefully thank Jasmin Jamal El-Din for her excellent technical assistance and intellectual input as well as Dana Wenzel for her outstanding support in the production of cell-free lysates. The authors additionally thank ACDBio technical support scientists Tijana Vujasinovic and Melissa Turan for troubleshooting assistance. T.H. is a member of the Clinician Scientist program funded by the 10.13039/501100002839Charité Universitätsmedizin Berlin and 10.13039/501100017268Berlin Institute of Health.

## Author contributions

T.H. conceptualized and established the protocol, created the figures, and wrote the manuscript. A.Z. synthesized cell-free lysates and *in vitro*-transcribed mRNA. A.M. supervised the study. All authors discussed the results and edited the manuscript.

## Declaration of interests

Author A.M. is a co-inventor on a previously filed but now abandoned patent (US20190106470A1) concerning the discovery of the EPO splice variant hS3.

## Declaration of generative AI and AI-assisted technologies in the writing process

During the preparation of this work, the author(s) used ChatGPT (GPT-5, OpenAI) in order to improve the clarity, grammar, and style of the English language. After using this tool/service, the author(s) reviewed and edited the content as needed and take(s) full responsibility for the content of the published article.

## References

[1] Meisel A.,P.J., Bonnas C., Dirnagl U. (2006). Erythropoietin Variants. Patent Application 12/334,995.

[2] Baker A.M., Huang W., Wang X.M.M., Jansen M., Ma X.J., Kim J., Anderson C.M., Wu X., Pan L., Su N. (2017). Robust RNA-based in situ mutation detection delineates colorectal cancer subclonal evolution. Nat. Commun..

[3] Bunda A., LaCarubba B., Akiki M., Andrade A. (2019). Tissue- and cell-specific expression of a splice variant in the II-III cytoplasmic loop of Cacna1b. FEBS Open Bio.

[4] Erben L., Buonanno A. (2019). Detection and Quantification of Multiple RNA Sequences Using Emerging Ultrasensitive Fluorescent In Situ Hybridization Techniques. Curr. Protoc. Neurosci..

[5] Zemella A., Thoring L., Hoffmeister C., Šamalíková M., Ehren P., Wüstenhagen D.A., Kubick S. (2018). Cell-free protein synthesis as a novel tool for directed glycoengineering of active erythropoietin. Sci. Rep..

[6] Dondapati S.K., Stech M., Zemella A., Kubick S. (2020). Cell-Free Protein Synthesis: A Promising Option for Future. BioDrugs.

[7] Kirby E., Tse W.H., Patel D., Keijzer R. (2019). First steps in the development of a liquid biopsy in situ hybridization protocol to determine circular RNA biomarkers in rat biofluids. Pediatr. Surg. Int..

[8] Cox L., Bai C., Platnich C.M., Rizzuto F.J. (2024). Divergent Polymer Superstructures from Protonated Poly(adenine) DNA and RNA. Biomacromolecules.

